# Effects of Thinning and Water Supply Manipulation on the Productivity of *Pinus sylvestris* var. *mongolica* in Northeastern China

**DOI:** 10.1371/journal.pone.0166109

**Published:** 2016-11-09

**Authors:** Yi Tang, Ming-yu Liu, Jin-hua Wu

**Affiliations:** School of Life Sciences, Liaoning University, Shenyang, China; University of California Davis, UNITED STATES

## Abstract

Management is an effective tool for increasing the productivity of Mongolian pine (*Pinus sylvestris* var. *mongolica*). This species has been widely planted in China, especially in sandy lands. However, optimization of management practices had not been fully explored. We established a system dynamic model to evaluate the effects of thinning and of manipulation of water supply on the productivity and population density of a Mongolian pine forest (17 scenarios in total). Different levels of thinning increased the mean biomass of Mongolian pine over no-management to a range from 202 to 256 t·ha^-1^. Increasing water supply decreased the mean biomass of Mongolian pine to a range from 176 to 199 t·ha^-1^. These results indicated that thinning at different levels may lead to an increase in biomass accumulation, while manipulating water supply may decrease biomass. Further, thinning appeared more effective than increasing water supply in efforts at maintaining high productivity of Mongolian pine forests. Moreover, the highest biomass occurred in a scenario with a thinning intensity of 30% in over-mature trees, indicating that this thinning intensity was the most effective choice for to the maintenance of the highest biomass in Mongolian pine forests. This study informs about the interactions between Mongolian pine and forest management, and provides guidelines for the practice of management of this forest type.

## 1 Introduction

Mongolian pine (*Pinus sylvestris* var. *mongolica*), a geographical variety of Scotch pine (*Pinus sylvestris* Linnaeus), has been widely planted in many parts of China, especially in sandy lands[1–3]. Because Mongolian pine is cold-tolerant, drought-resistant, and adapts to a broad range of conditions, it develops into a main tree species on sandy lands [4]. The area of Mongolian pine in sand lands exceeds 3.0×10^5^ ha in northern China [1, 3].

Mongolian pine plantations exhibit symptoms of decline, including dead stems, wilting of the upper plant parts, and low growth rates [1, 5]. Healthy Mongolian pine stands could play an important role in combating desertification and promoting excellent ecological and social benefits to sandy lands [6]. Therefore, management goals for Mongolian pine plantations that incorporate reversing of the degradation and promoting of further development have received increased attention [3].

Stand thinning practices are an effective way to promote tree growth and to accelerate productivity [7]. Thinning may also increase ambient temperatures in the stands, improve light conditions, and promote the growth of understory vegetation [8]. In practice, stand thinning is a critical tool for regulating the density of Mongolian pine plantations and improving economic benefits [9]. However, the effects of thinning on Mongolian pine productivity are not fully understood, making optimization of thinning practices not possible. Jiang [10] observed that the growth of Mongolian pine plantations is below expectation with high and with low thinning levels, and You et al. [11] related mean diameter and density in artificial Mongolian pine forest; however, the most effective thinning intensity and stand age are unknown. Increasing the knowledge of thinning effects is critically important for guiding the silviculture of Mongolian pine forests.

Most of the earlier studies were based on empirical methods. Such methods may be limited to short-term observations and to expensive labor- and experiment-costs [12,13]. Simulation methods are a feasible way of evaluating effects of thinning strategies over long time, and large spatial scales [14]. A system dynamic (SD) model can simulate the dynamics of populations using a special semiotic system to express the relationships between variables [15]. For example, a SD model was used to simulate the conversion processes of nitrogen, and to verify the threshold value of regional eco-security [16,17].

In arid and semi-arid lands, drought in the early 1990's may have been a major cause of death of Mongolian pine over large areas [5,18,19]. Increasing water supply is helpful for promoting growth of Mongolian pine and for maintaining population stability [20]. However, the relationship between increasing water supply and productivity of Mongolian pine plantations is not well known. A greater understanding of the interactions between plants and water in sandy lands is critical for guiding the management of Mongolian pine forests.

The main aims of this study were to understand the productivity responses of the Mongolian pine forest to: (1) different intensity of thinning, (2) several stand thinning stages, and (3) increasing water supply.

To address these aims, we constructed a system dynamic model with population density and biomass modules, which are the two status variables in this model. The thinning and water supply treatments were included separately as auxiliary variables in the study. The results of this study will increase the understanding of the effects of management on productivity of the Mongolian pine forest.

## 2 Materials and Methods

### 2.1 Building of the system dynamic model

**Fig 1:**
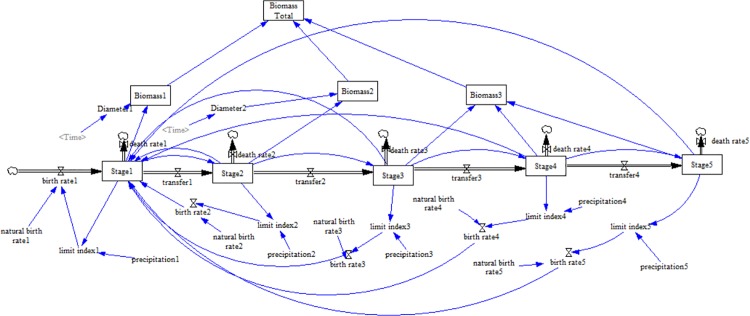
A stock-flow diagram representing the simulated system.

The stock-flow diagram is shown in Fig 1. The system dynamic model was established following the equations below.

$$\begin{array}{l} \frac{dN_{i}}{dt}=\sum_{i=1}^{n}B_{i}\cdot N_{i}-(D_{i}+TP_{i})\cdot N_{i}\cdots \cdots \cdots \cdots \cdots \text{for stage}\;1\\ \frac{dN_{i}}{dt}=TP_{i-1}\cdot N_{i-1}-(D_{i}+TP_{i})\cdot N_{i}\cdots \cdots \cdots i=2,\cdots ,n-1\\ \frac{dN_{i}}{dt}=TP_{i-1}\cdot N_{i-1}-D_{i}\cdot N_{i}\cdots \cdots \cdots \cdots \cdots \cdots \cdots \cdots \cdots i=n\\ Bio=\sum_{i=1}^{n}bio_{i}\cdot N_{i}\cdot \cdots \cdots \cdots \cdots \cdots \cdots \cdots \cdots \cdots \text{for stage}\;1-5 \end{array}$$

where N was the population size, D was death rate, i.e. the number of deaths individuals per 1 individual in a stage, TP was the transition probability (i.e. probability associated with a pine population at one age-stage shifting to another age-stage), i indicated stages from 1 to 5, B was birth rate. Bio was the biomass of the population, bio was the biomass of the individuals. The biomass of individuals (bio) could be expressed as a function of its diameters (d).

Thinning and manipulation of water supply treatments were incorporated into the SD model separately. Thinning levels were represented by a pulse-train function, and manipulation of water supply as a step function [21]. Manipulation of water supply in this study is regulating the parameter referred to as precipitation.

### 2.2 Parameters settings

This study was conducted in Zhanggutai region (42°35′-42°47′N, 122°23′-122°40′E, altitude 226.5 m), which is located in the southern part of the Horqin Sandy Land. In this region, Mongolian pine was planted from the 1950's until present [5]. This region belongs to typical semi-arid climatic conditions and more than 60% of precipitation falls during the months from June to August. The lowest monthly mean temperature is -12.5°C(January) and the highest monthly mean temperature is 23.8°C(July). The soil is an eolian sandy type and three main types of land use are coniferous forests, broadleaved forests and dry fields [22].Diameters at breast height (1.3 m) of individual trees aged less than 30 years were obtained by measuring 4047 individuals with the diameter tape [23]. Diameters (d) of individual trees aged more than 30 years were obtained from equation d = 2180·N^-0.6614^, where N was the population density [24]. We developed an equation to express the relationship between individual-tree biomass (bio) and its diameter (d), i.e., bio = 0.051·d^2.595^, P<0.05, R^2^ = 0.989), based on observation data [25]. The five stages of Mongolian pine populations were defined based on age ranges of the forest, i.e. young: 1–20 years, half-mature: 21–30 years, near-mature: 31–40 years, mature: 41–60 years, and over-mature forest: 61–150 years old [26].The values of other parameters were reported in a previous study [26]. The initial density of Mongolian pine is 10,000 young trees per hectare at the beginning of simulation. To evaluate the effects of the thinning treatments, we simulated a thinning regime in which the forest was thinned once every 10 years for 150 years, with stable age structure; this accommodated life history of Mongolian pine which is 150 years.

### 2.3 Model validation and simulation

We used unit-consistency test to validate this model. The unit-consistency test, which checks for agreement among units, was automatically completed in the Vensim package [27]. The model was formulated and simulated using a professional SD software package 'Ventana Simulation Environment Personal Learning Edition (Vensim PLE)'. The simulation was run for 1000 steps, and a modeling step was 1 year.

### 2.4 Scenario analysis

We evaluated the effects of different water-supply levels. The percent of water supply increase considered in scenarios was 5 (corresponding to additional 25 mm of annual precipitation), 10 (additional 50 mm of annual precipitation), and 20 (additional 100 mm of annual precipitation), tested separately. Additionally, we tested the scenario of maintaining precipitation at 500 mm (called 500 mm of precipitation from now on). We also evaluated the effects of different thinning intensities. The thinning treatments were tested in age-groups of over-mature, mature, and juvenile trees, separately. The thinning intensities were 5, 10, 20, and 30% of each age group. For instance, thinning intensity of 5% in mature trees meant that stems from a total of 5% of the area in mature trees were removed. In total, 17 scenarios were analyzed, including one without any treatment (reference).

## 3 Results

### 3.1 The effects of water supply treatments

**Fig 2:**
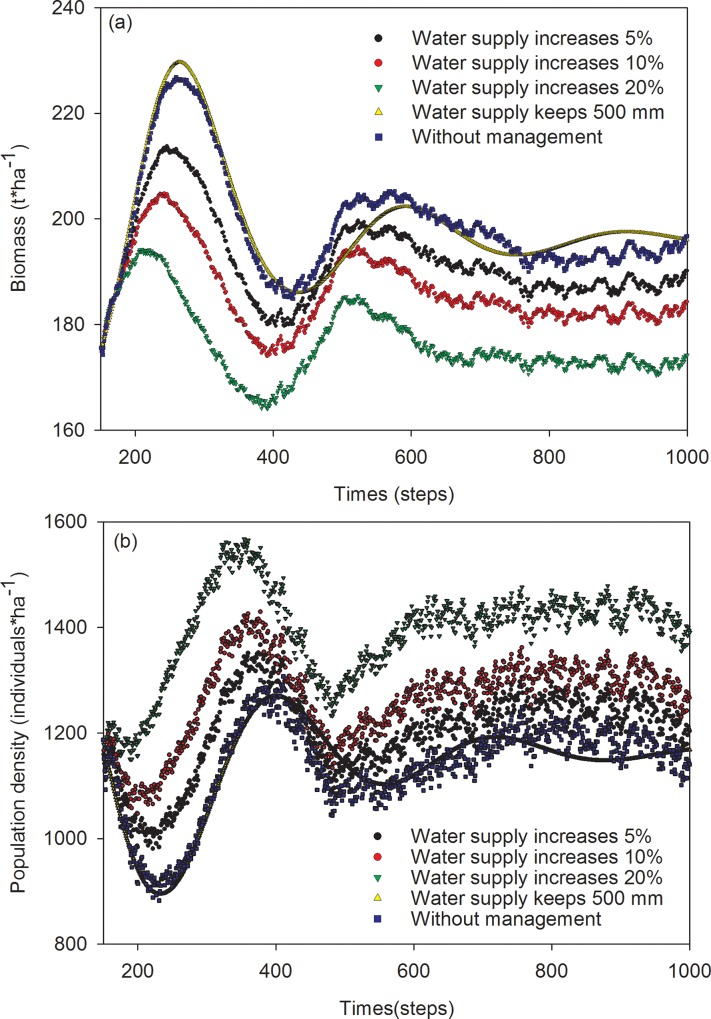
Simulation results of the effects of increasing water supply on biomass (a) and population density (b) of Mongolian pine forest.

The mean biomass of Mongolian pine in simulating periods ranged from 176 to 199 t·ha^-1^ across five scenarios, i.e. water supply of 5, 10, and 20%, maintaining 500 mm of precipitation, and without management. The highest mean biomass occurred in two scenarios, i.e., without management, and maintaining 500 mm of precipitation. The lowest mean biomass occurred in scenario with water supply at 20%. The mean biomass was 186 and 192 t·ha^-1^ in scenarios with water supply of 10 and 5%, respectively. The order of mean biomass descended as follows: maintaining 500 mm of precipitation > without management > water supply of 5% > water supply of 10% > water supply of 20%. The mean biomass of Mongolian pine in scenarios without management and maintaining 500 mm were equal. Fluctuations of biomass in scenario maintaining 500 mm of precipitation were smaller than those without management (Fig 2A).

The population density of Mongolian pine in simulating periods ranged from 1134 to 1391 individuals· ha^-1^ across five water supply scenarios. The order of population densities in scenarios descended as follows: water supply at 20% > 10% > 5% > without management > maintaining 500 mm of precipitation. The fluctuation of population densities in scenario maintaining 500 mm of precipitation was the smallest in these five scenarios (Fig 2B).

### 3.2 The effects of thinning treatments

**Fig 3:**
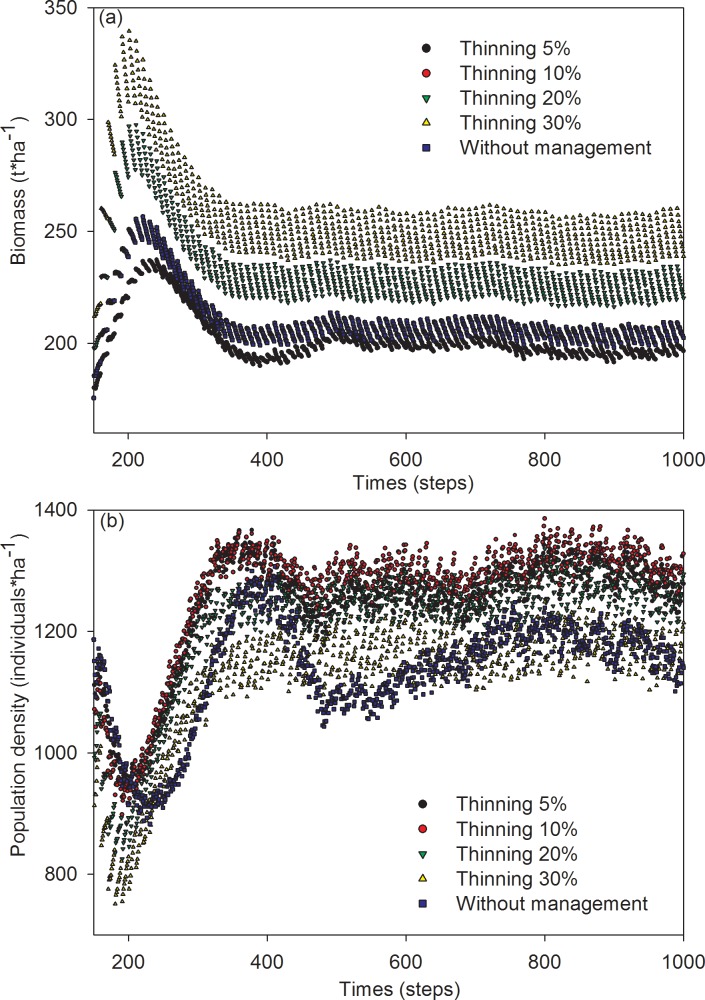
Simulation results of the effects of thinning on biomass (a) and population density (b) of over mature trees in Mongolian pine forest.

In scenarios, in which thinning was performed on over-mature trees, the mean biomass of Mongolian pine ranged from 202 to 256 t·ha^-1^. The order of mean biomass descended across thinning-intensity scenarios as follows: 30% > 20% > 10% > 5% (Fig 3A). Meanwhile, the population density of Mongolian pine ranged from 1120 to 1263 individuals·ha^-1^. The order of population density descended across thinning-intensity scenarios as follows: 10% > 5% > 20% > 30% (Fig 3B).

**Fig 4:**
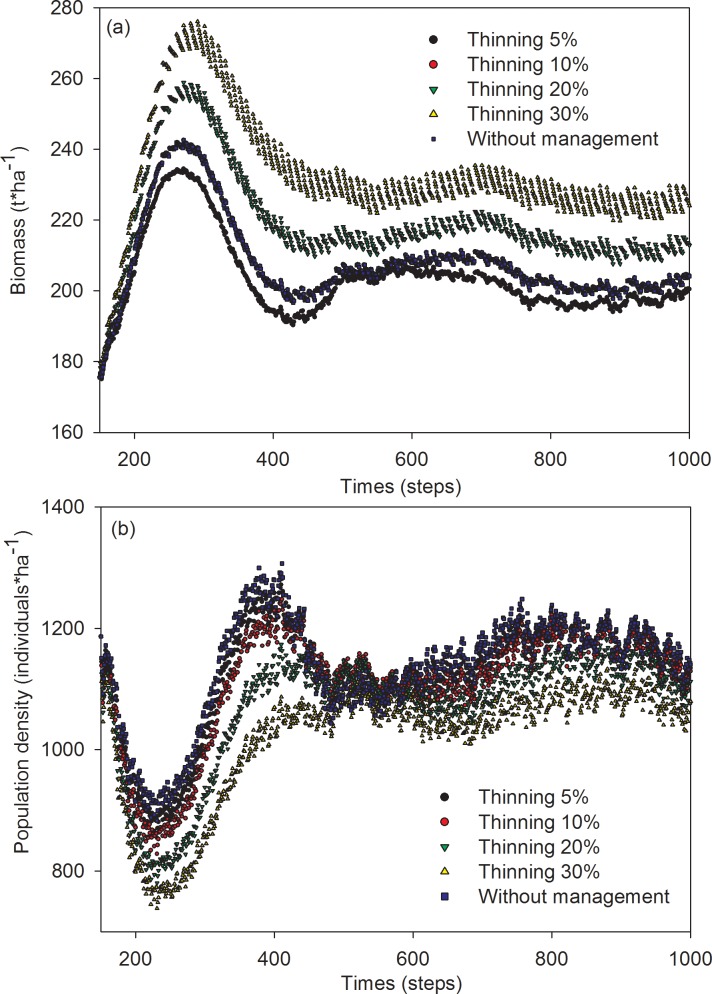
Simulation results of the effects of thinning on biomass (a) and population density (b) of mature trees in Mongolian pine forest.

In scenarios, in which thinning was tested on mature trees, the mean biomass of Mongolian pine ranged from 203 to 233 t·ha^-1^. The order of mean biomass descended across thinning-intensity scenarios as follows: 30% > 20% > 10% > 5% (Fig 4A). Meanwhile, the population density of Mongolian pine ranged from 1017 to 1122 individuals· ha^-1^. The order of population density descended across thinning-intensity scenarios as follows 5% > 10% > 20% > 30% (Fig 4B).

**Fig 5:**
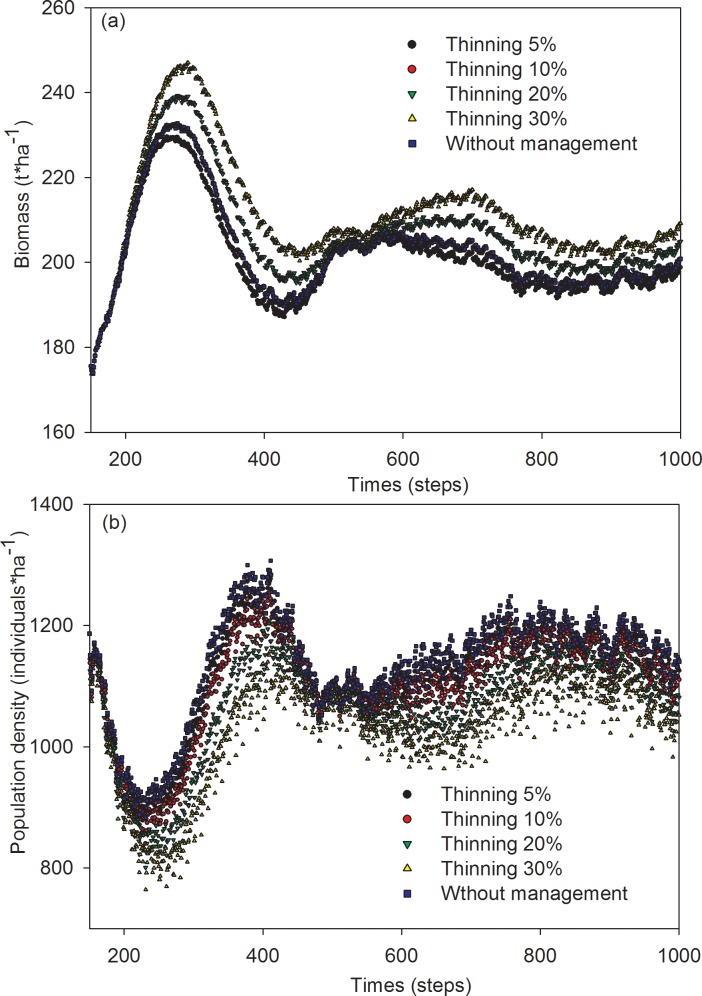
Simulation results of the effects of thinning on biomass (a) and population density (b) of juvenile trees in Mongolian pine forest.

The mean biomass of Mongolian pine in simulation periods ranged from 201 to 212 t·ha^-1^ for thinning treatments with juvenile trees. The order of mean biomass descended across thinning-intensity scenarios as follows: 30% > 20% > 10% > 5% (Fig 5A). Meanwhile, the population density of Mongolian pine ranged from 1025 to 1117 individuals· ha^-1^. The order of population density descended across thinning-intensity scenarios as follows: 5% > 10% > 20% > 30% (Fig 5B).

## 4 Discussion

According to our results, the highest simulated biomass was obtained with thinning intensity of 30% in over-mature trees, indicating that this combination of thinning level and tree-age group may be the best choice of treatment for maintaining the highest biomass in Mongolian pine forests. These results were consistent with an experimental study, where the biomass of a Mongolian pine forest thinned at 30% was greater than that obtained with other thinning intensities, (i.e., thinning intensity 10, 20, or 40%) [28]. Additionally, the biomass of our Mongolian pine forest following thinning treatments was greater than that without thinning, indicating that thinning promotes the accumulation of biomass. The accumulation of biomass is closely related to the growth of Mongolian pine trees and their habitats. Thinning could play an important role in promoting the growth of Mongolian pine trees, and in regulating the micro-environment in Mongolian pine forests [29].

The mean biomass of the Mongolian pine forest in our study following water supply treatments ranged from 176 to 192 t·ha^-1^, which was less than that without treatment. This indicated that increasing water supply was not effective in maintaining high biomass of Mongolian pine forest in the Horqin Sandy Land. In the Horqin Sandy Land, an increase in water supply might not increase the growth rate of Mongolian pine, and may not significantly affect its eco-physiological characteristics, such as soluble protein, malondialdehyde content, and Superoxide Dismutase activity [30]. These results are consistent with those of a previous study, where *Encelia canescens* in arid environments exhibited reductions in biomass to cope with low water availability [31].

The population of the Mongolian pine forest in this study ranged from 1201 to 1391 individuals·ha^-1^ in water supply simulations, and was 1134 individuals ·ha^-1^ without treatment, indicating that increasing the water supply could expand the population density in this forest.

Thinning treatments in this forest type had both positive and negative effects on population density, depending on tree age. Thus, thinning of over-mature trees had mostly positive effects, while thinning of mature and juvenile trees had negative effects on population densities of this Mongolian pine forest. We conclude that the age-structure should be considered in evaluating the potential effectiveness of thinning practices on the population density of a Mongolian pine forest.

This study revealed management trade-offs between targeting biomass growth and population density. A management strategy promoting the accumulation of biomass will decrease population density. In contrast, a management strategy aimed at increasing population density will decrease the accumulation of biomass. In general, practices that amend water supply may lead to an increase in population density and a decrease in biomass accumulation. Meanwhile, thinning treatments may result in increases in biomass and decreases in population density. Thus, to maintain high productivity of Mongolian pine forests, thinning practices appear more effective than manipulations of water supply. Trade-offs between size and biomass at an individual-tree level, which are influenced by environmental factors or disturbances, have also been reported [32–34].

In this model, the biomass of Scots pine is mainly determined by water supply, which is the same as in a previous study [35]. According to the results simulated in this model, the mean biomass of over-mature stands thinned to 30% was 256 t·ha^-1^ in the simulation period; this approximated the results of a previous study in Scots pine stands on sandy soils, with biomass of 258.4 t·ha^-1^ post thinning and sanitary fellings [36]. According to Cienciala’s study, biomass of 50- and 100-year-old Scots pine were 156.0 and 261.9 t·ha^-1^ more than that in this study [37]. The difference may be due to the different precipitation. The precipitation in Zhanggutai region is less than 500 mm in mean annual precipitation. In the Cienciala’s study area, the mean annual precipitation is more than 500 mm generally [38].

A system dynamic model was used here to simulate the biomass and population density of the Mongolian pine forest. We considered the influence of environmental factors and management practices on population characteristics of Mongolian pine. The model is a mathematical representation of key environmental factors and management approaches and makes estimates of physiological and ecological parameters, which could enhance our understanding of the ecological mechanisms critical for the growth of Mongolian pine populations. In addition to the SD model used here, several other models are used to simulate the biomass of plant populations. For example, the 3-PG process-based model is used to simulate the biomass growth under thinning management [7]. Unlike the 3-PG model, which incorporates an understanding of physiological and ecological mechanisms, the SD model does not need to consider complex processes; this makes the model more user-friendly. For the specific conditions represented in this study, where the interaction of plant and environmental factors was not well known, the SD model was relatively simple and easy to apply.

The effects of thinning on biomass are mainly considered in this study. However, thinning treatments could influence forests in other aspects. For example, thinning treatments could increase the throughfall, change the static stability and increase the light-use efficiency [39,40]. Besides biomass, changes of Mongolian pine populations caused by thinning treatments should be considered in further studies.

In conclusion, the highest biomass, of 256 t·ha^-1^, in the Mongolian pine forest in this study, occurred in the 30% thinning treatment in over-mature trees. We conclude that such combination of thinning and tree age is the most effective tool for maintaining the highest biomass in the Mongolian pine forest in the Horqin Sandy Land. Moreover, manipulation of water supply in this forest type in this area may lead to an increase in population density and a decrease in biomass accumulation. Meanwhile, thinning may lead to an increase in biomass accumulation and a decrease in population density. We suggest that thinning is more effective than increasing water supply in maintaining high productivity of this Mongolian pine forest. The results of this study inform about the interaction between Mongolian pine and management practices, and can serve as guidelines for the management of the Mongolian pine forest.
